# Supplementary material for: Content-rich biological network constructed by mining PubMed abstracts
Source: BMC Bioinformatics. 2004 Oct 8;5:147. doi: 10.1186/1471-2105-5-147 (PMC528731; doi:10.1186/1471-2105-5-147)
Supplement: Additional File 5 — The original Chilibot query results of the term "long-term potentiation (LTP)" and 22 other terms, limiting the latest references analyzed to the years 1990, 1995, 2000, and 2004. [file 1471-2105-5-147-S5.bz2 › chilibotAdditionalFile5/ltp2004/html/ERK.html]

 


**ERK** (Input: ERK ) 

---


**Google Searches:** Entire Web  | EDU domain only  | PDF files only

.

**External Links:** OMIM |
LocusLink
| Swissprot
| GeneCards

  
**Maps of ERK**

Simple
Complete
graph in
radiant
tree
square
layout.

**New Hypothesis !**

**Synonyms** 

- erk   [PubMed]

**Synopsis**

- **ERK** is required for the early phase of LTP elicited by brief 5 Hz stimulation, as well as for LTP elicited by more prolonged 5 Hz stimulation when paired with beta1 adrenergic receptor activation.  Neuron, 1999 
  [22]

- Glutamate release was enhanced, and the data suggest that this may be a consequence of increased activation of TrkB and **ERK**.  Behav Neurosci, 2002 
  [22]

- Our results reveal an unexpected complexity of **ERK** dependent signaling in the brain and a critical regulatory role for ERK1 in the long term adaptive changes underlying striatum dependent behavioral plasticity and drug addiction.  Neuron, 2002 
  [20]

- We show that pharmacological blockade of MAPK **ERK** phosphorylation, functional inactivation of CREB in an inducible transgenic mouse and inactivation of zif268 in a mutant mouse result in a similar deficit in long term recognition memory.  Philos Trans R Soc Lond B Biol Sci, 2003 
  [20]

- These changes are accompanied by increased activation of one member of the family of mitogen activated protein kinases, extracellular signal regulated kinase **ERK** and the data indicate that these events play a role in modulating release of glutamate from perforant path granule cell synapses, because the Trk inhibitor K252a and the **ERK** inhibitor, UO126, both inhibited the BDNF induced enhancement of release.  J Neurochem, 2001 
  [20]

- However, the individual roles for the two isoforms of **ERK** have yet to be deciphered.  Learn MemLearn Mem, 2001 
  [19]

- The NO cGMP PKG **ERK** signaling pathway thus plays important role in the potentiation of LTP by YC 1.  Mol Pharmacol, 2003 
  [19]

- Thus, we further show that, in both PC12 cells and hippocampal neurons, depolarization induced calcium influx stimulates **ERK** activity in a PKA dependent manner.  J Biol Chem, 2000 
  [19]

- Together, our results indicate that the role of PI3 kinase in LTP is NOT limited to its role as an upstream regulator of MAPK signaling but includes signaling through **ERK** independent pathways that regulate LTP induction.  J Neurosci, 2003 
  [17]

- Although cAMP may be required for several events in this complex signal transduction cascade, one of the major roles of cAMP may be to support nuclear translocation of **Erk** MAP kinase in hippocampal neurons.  Int J Dev Neurosci, 2001 
  [16]

- Thus, MAPK **ERK** activation is required for LTP dependent transcriptional regulation and we suggest this is regulated by two parallel signaling pathways, the MAPK **ERK** Elk 1 pathway targeting SRE and the MAPK **ERK** CREB pathway targeting CRE.  J Neurosci, 2000 
  [16]

- Given the possible role of altered gene expression in the late phase of LTP and in long term memory, we evaluated the capacity of the MAPK **ERK** extracellular signal regulated kinase to regulate phosphorylation of the transcription factor cAMP response element binding protein CREB in hippocampal area CA1.  Acta Neurobiol Exp (Wars), 2000 
  [16]

- Overall, our data indicate that the progressive increase in spiking observed during TBS represents a form of physiologic temporal integration that is dependent on **ERK** MAPK activity.  Learn MemLearn Mem, 2000 
  [14]

- Collectively, these results demonstrate that **ERK** MAPK activation is necessary for both memory consolidation of Pavlovian fear conditioning and synaptic plasticity in the amygdala.  J Neurosci, 2000 
  [14]

- These results demonstrate that the **ERK** pathway is necessary for experience dependent plasticity and for LTP of synaptic transmission in the developing visual cortex.  Science, 2001 
  [14]
